# {4,4′-Dimethyl-2,2′-[(2,2-dimethyl­propane-1,3-di­yl)bis­(nitrilo­methanylyl­idene)]diphenolato}nickel(II) monohydrate

**DOI:** 10.1107/S1600536811054262

**Published:** 2011-12-23

**Authors:** Hadi Kargar, Reza Kia, Zahra Sharafi, Muhammad Nawaz Tahir

**Affiliations:** a Department of Chemistry, Payame Noor University, PO Box 19395-3697 Tehran, Iran; b X-ray Crystallography Lab., Plasma Physics Research Center, Science and Research Branch, Islamic Azad University, Tehran, Iran; c Department of Chemistry, Science and Research Branch, Islamic Azad University, Tehran, Iran; d Department of Chemistry, Marvdasht Branch, Islamic Azad University, Marvdasht, Iran; e Department of Physics, University of Sargodha, Punjab, Pakistan

## Abstract

In the title compound, [Ni(C_21_H_24_N_2_O_2_)]·H_2_O, both the complex mol­ecule and the water mol­ecule lie on a twofold rotation axis. The Ni^II^ ion is coordinated in a distorted square-planar geometry by the tetra­dentate ligand. The dihedral angle between the two symmetry-related benzene rings is 47.12 (8)°. In the crystal, pairs of symmetry-related O—H⋯O hydrogen bonds form *R*_2_^2^(6) ring motifs. In addition, there are weak inter­molecular C—H⋯O hydrogen bonds, and π–π stacking inter­actions with a centroid–centroid distance of 3.4760 (8) Å.

## Related literature

For related structures, see for example: Fun *et al.* (2008 ▶); Kargar *et al.* (2008 ▶, 2011 ▶); Rayati *et al.* (2011 ▶); Kia *et al.* (2010 ▶). For standard bond lengths, see: Allen *et al.* (1987 ▶). For hydrogen-bond motifs, see: Bernstein *et al.* (1995 ▶).
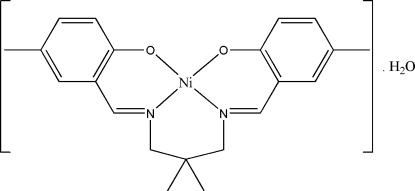


## Experimental

### 

#### Crystal data

[Ni(C_21_H_24_N_2_O_2_)]·H_2_O
*M* *_r_* = 413.15
Monoclinic, 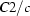

*a* = 13.3333 (4) Å
*b* = 15.9424 (5) Å
*c* = 9.9965 (3) Å
β = 104.736 (1)°
*V* = 2055.01 (11) Å^3^
*Z* = 4
Mo *K*α radiation
μ = 0.97 mm^−1^
*T* = 296 K
0.25 × 0.12 × 0.08 mm

#### Data collection

Bruker SMART APEXII CCD area-detector diffractometer
Absorption correction: multi-scan (*SADABS*; Bruker, 2005 ▶) *T*_min_ = 0.794, *T*_max_ = 0.927
17468 measured reflections
2557 independent reflections
2131 reflections with *I* > 2σ(*I*)
*R*_int_ = 0.040

#### Refinement

*R*[*F* ^2^ > 2σ(*F* ^2^)] = 0.031
*wR*(*F* ^2^) = 0.083
*S* = 1.06
2557 reflections
125 parameters
H-atom parameters constrained
Δρ_max_ = 0.21 e Å^−3^
Δρ_min_ = −0.31 e Å^−3^

### 

Data collection: *APEX2* (Bruker, 2005 ▶); cell refinement: *SAINT* (Bruker, 2005 ▶); data reduction: *SAINT*; program(s) used to solve structure: *SHELXTL* (Sheldrick, 2008 ▶); program(s) used to refine structure: *SHELXTL*; molecular graphics: *SHELXTL*; software used to prepare material for publication: *SHELXTL*.

## Supplementary Material

Crystal structure: contains datablock(s) global, I. DOI: 10.1107/S1600536811054262/lh5396sup1.cif

Structure factors: contains datablock(s) I. DOI: 10.1107/S1600536811054262/lh5396Isup2.hkl

Additional supplementary materials:  crystallographic information; 3D view; checkCIF report

## Figures and Tables

**Table 1:** Hydrogen-bond geometry (Å, °)

*D*—H⋯*A*	*D*—H	H⋯*A*	*D*⋯*A*	*D*—H⋯*A*
O1*W*—H1⋯O1^i^	0.98	1.92	2.781 (2)	145
C3—H3*A*⋯O1*W*^ii^	0.93	2.55	3.477 (2)	173

Symmetry codes: (i) 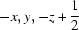; (ii) 
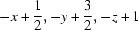
.

**Fig. 1.**
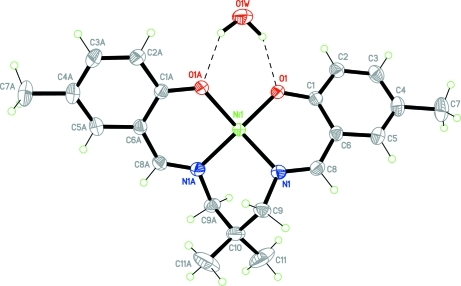
The molecular structure of the title compound, showing 40% probability displacement ellipsoids. The dashed lines show hydrogen bonds [symmetry code: (A) -x, y, -z+1/2].

**Fig. 2.**
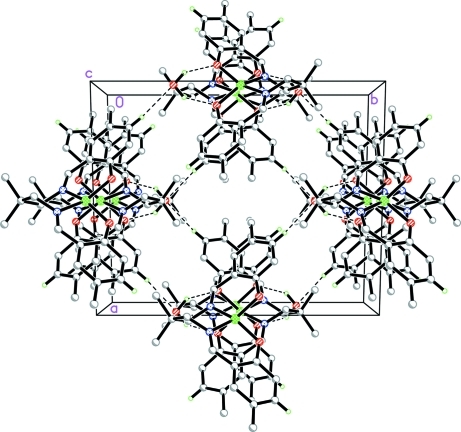
A partial packing diagram of the title compound viewed approximately along the c-axis showing molecules linked through intermolecular hydrogen bonds (dashed lines). Only the H atoms involved in the interactions are shown.

## supplementary crystallographic information

### Comment

In continuation of our work on the crystal structures of a Schiff base ligands and complexes (Fun *et al.*, 2008; Kargar *et al.*, 2008,2011; Rayati *et al.,* 2011; Kia *et al.,*  2010), we have determined the X-ray structure of the title compound.

The molecular structure of the title compound is ahown in Fig. 1. The asymmetric unit comprises half of Schiff base complex and half a water molecule. The Ni^II^ ion, the central carbon atom of the diamine segment (C10) and the O atom of water molecule lie on a two-fold rotation axis. The coordination geometry of Ni1 is distorted square-planar formed by the tetradentate ligand. The bond lengths (Allen *et al.,* 1987) and angles are within the normal ranges and are comparable to related structures (Fun *et al.* 2008; Kargar *et al.* 2008; Rayati *et al.,* 2011). The dihedral angle between the two symmetry related benzene rings is 47.12 (8)°. A pair of symmetry related intermolecular O—H···O hydrogen bonds form an R^2^_2_(6) ring motif (Bernstein *et al.*, 1995).  In the crystal, molecules are linked through weak intermolecular C—H···O interactions. The crystal structure is further stabilized by intermolecular π–π interactions [*Cg*1···*Cg*1^iii^ = 3.4760 (8)Å; (iii) -*x*, 1 - *y*, 1 - *z*; *Cg*1 is the centroid of the Ni1/O1/C1/C6/C8/N1 ring].

### Experimental

The title compound was synthesized by adding bis(5-methylsalicylaldehyde)-2,2-dimethyl-1,3-propanediimine (2 mmol) to a solution of nickel(II) chloride hexahydrate (2.1 mmol) in ethanol (30 ml). The mixture was refluxed with stirring for half an hour. The resultant solution was filtered. Yellow single crystals of the title compound suitable for *X*-ray structure determination were recrystallized from an ethanol solution of the title compound by slow evaporation of the solvent at room temperature over several days.

### Refinement

Hydrogen atoms bonded to C atoms were positioned geometrically with C—H = 0.93-0.97 Å and included in a riding model approximation with U_iso_ (H) = 1.2 or 1.5 U_eq_ (C) The unique water H atom was located in a difference Fourier map and then constrained to ride to the parent atom with U_iso_ (H) = 1.5 U_eq_ (O). A rotating group model was used only for the benzene- substituent methyl group.

### Crystal data

**Table:**

[Ni(C_21_H_24_N_2_O_2_)]·H_2_O	*F*(000) = 872
*M**_r_* = 413.15	*D*_x_ = 1.335 Mg m^−^^3^
Monoclinic, *C*2/*c*	Mo *K*α radiation, λ = 0.71073 Å
Hall symbol: -C 2yc	Cell parameters from 3245 reflections
*a* = 13.3333 (4) Å	θ = 2.8–27.8°
*b* = 15.9424 (5) Å	µ = 0.97 mm^−^^1^
*c* = 9.9965 (3) Å	*T* = 296 K
β = 104.736 (1)°	Block, red
*V* = 2055.01 (11) Å^3^	0.25 × 0.12 × 0.08 mm
*Z* = 4	

### Data collection

**Table:**

Bruker SMART APEXII CCD area-detector diffractometer	2557 independent reflections
Radiation source: fine-focus sealed tube	2131 reflections with *I* > 2σ(*I*)
graphite	*R*_int_ = 0.040
φ and ω scans	θ_max_ = 28.3°, θ_min_ = 2.6°
Absorption correction: multi-scan (*SADABS*; Bruker, 2005)	*h* = −17→16
*T*_min_ = 0.794, *T*_max_ = 0.927	*k* = −21→21
17468 measured reflections	*l* = −13→13

### Refinement

**Table:**

Refinement on *F*^2^	Primary atom site location: structure-invariant direct methods
Least-squares matrix: full	Secondary atom site location: difference Fourier map
*R*[*F*^2^ > 2σ(*F*^2^)] = 0.031	Hydrogen site location: inferred from neighbouring sites
*wR*(*F*^2^) = 0.083	H-atom parameters constrained
*S* = 1.06	*w* = 1/[σ^2^(*F*_o_^2^) + (0.0485*P*)^2^] where *P* = (*F*_o_^2^ + 2*F*_c_^2^)/3
2557 reflections	(Δ/σ)_max_ < 0.001
125 parameters	Δρ_max_ = 0.21 e Å^−^^3^
0 restraints	Δρ_min_ = −0.31 e Å^−^^3^

### Special details

**Table:**

Geometry. All esds (except the esd in the dihedral angle between two l.s. planes) are estimated using the full covariance matrix. The cell esds are taken into account individually in the estimation of esds in distances, angles and torsion angles; correlations between esds in cell parameters are only used when they are defined by crystal symmetry. An approximate (isotropic) treatment of cell esds is used for estimating esds involving l.s. planes.
Refinement. Refinement of F^2^ against ALL reflections. The weighted R-factor wR and goodness of fit S are based on F^2^, conventional R-factors R are based on F, with F set to zero for negative F^2^. The threshold expression of F^2^ > 2sigma(F^2^) is used only for calculating R-factors(gt) etc. and is not relevant to the choice of reflections for refinement. R-factors based on F^2^ are statistically about twice as large as those based on F, and R- factors based on ALL data will be even larger.

### Fractional atomic coordinates and isotropic or equivalent isotropic displacement parameters (Å^2^)

**Table:**

	*x*	*y*	*z*	*U*_iso_*/*U*_eq_	
Ni1	0.0000	0.489837 (15)	0.2500	0.03589 (11)	
O1	0.09186 (9)	0.57316 (6)	0.34800 (11)	0.0457 (3)	
N1	0.04523 (10)	0.40475 (8)	0.39178 (12)	0.0414 (3)	
C1	0.16634 (12)	0.56212 (9)	0.46203 (15)	0.0401 (3)	
C2	0.23779 (14)	0.62692 (10)	0.50786 (17)	0.0497 (4)	
H2A	0.2346	0.6747	0.4537	0.060*	
C3	0.31279 (14)	0.62162 (12)	0.63132 (18)	0.0567 (5)	
H3A	0.3589	0.6660	0.6581	0.068*	
C4	0.32147 (14)	0.55163 (13)	0.71719 (17)	0.0550 (4)	
C5	0.25490 (14)	0.48675 (11)	0.67168 (18)	0.0487 (4)	
H5A	0.2604	0.4390	0.7265	0.058*	
C6	0.17758 (13)	0.48878 (9)	0.54427 (17)	0.0410 (3)	
C7	0.40114 (17)	0.54821 (18)	0.85526 (19)	0.0817 (7)	
H7A	0.4556	0.5101	0.8494	0.123*	
H7B	0.3687	0.5292	0.9252	0.123*	
H7C	0.4297	0.6031	0.8788	0.123*	
C8	0.11388 (13)	0.41566 (10)	0.50691 (15)	0.0431 (4)	
H8A	0.1230	0.3725	0.5714	0.052*	
C9	−0.01669 (14)	0.32780 (10)	0.36982 (16)	0.0488 (4)	
H9A	−0.0895	0.3426	0.3512	0.059*	
H9B	0.0006	0.2949	0.4542	0.059*	
C10	0.0000	0.27358 (15)	0.2500	0.0564 (7)	
C11	0.0973 (2)	0.21930 (15)	0.2981 (2)	0.1038 (9)	
H11A	0.1569	0.2548	0.3287	0.156*	
H11B	0.1057	0.1851	0.2226	0.156*	
H11C	0.0903	0.1840	0.3729	0.156*	
O1W	0.0000	0.72531 (12)	0.2500	0.0992 (8)	
H1	−0.0556	0.6878	0.2007	0.149*	

### Atomic displacement parameters (Å^2^)

**Table:**

	*U*^11^	*U*^22^	*U*^33^	*U*^12^	*U*^13^	*U*^23^
Ni1	0.03715 (18)	0.03024 (16)	0.03666 (16)	0.000	0.00274 (11)	0.000
O1	0.0462 (7)	0.0353 (6)	0.0472 (6)	−0.0002 (5)	−0.0032 (5)	0.0020 (5)
N1	0.0448 (8)	0.0367 (7)	0.0424 (7)	−0.0023 (6)	0.0110 (6)	0.0012 (5)
C1	0.0364 (8)	0.0408 (8)	0.0418 (8)	0.0035 (6)	0.0075 (6)	−0.0040 (6)
C2	0.0470 (10)	0.0449 (9)	0.0540 (9)	−0.0035 (7)	0.0069 (8)	−0.0022 (7)
C3	0.0434 (10)	0.0635 (12)	0.0586 (10)	−0.0101 (8)	0.0047 (8)	−0.0118 (9)
C4	0.0391 (10)	0.0794 (13)	0.0432 (9)	−0.0003 (9)	0.0042 (7)	−0.0047 (9)
C5	0.0428 (10)	0.0604 (11)	0.0413 (8)	0.0055 (8)	0.0076 (7)	0.0051 (7)
C6	0.0373 (9)	0.0449 (9)	0.0390 (8)	0.0040 (7)	0.0065 (7)	−0.0010 (6)
C7	0.0590 (14)	0.121 (2)	0.0537 (11)	−0.0122 (13)	−0.0074 (10)	−0.0005 (12)
C8	0.0472 (10)	0.0411 (8)	0.0408 (8)	0.0029 (7)	0.0109 (7)	0.0055 (6)
C9	0.0603 (11)	0.0394 (9)	0.0494 (9)	−0.0103 (8)	0.0189 (8)	0.0006 (7)
C10	0.0806 (19)	0.0355 (12)	0.0563 (14)	0.000	0.0236 (14)	0.000
C11	0.159 (3)	0.0726 (15)	0.0929 (16)	0.0629 (16)	0.0553 (17)	0.0332 (13)
O1W	0.1069 (18)	0.0426 (11)	0.1201 (18)	0.000	−0.0225 (15)	0.000

### Geometric parameters (Å, °)

**Table:**

Ni1—O1	1.901 (1)	C5—H5A	0.9300
Ni1—O1^i^	1.9010 (10)	C6—C8	1.435 (2)
Ni1—N1^i^	1.9436 (12)	C7—H7A	0.9600
Ni1—N1	1.9436 (12)	C7—H7B	0.9600
O1—C1	1.3194 (17)	C7—H7C	0.9600
N1—C8	1.2870 (19)	C8—H8A	0.9300
N1—C9	1.4638 (19)	C9—C10	1.539 (2)
C1—C2	1.401 (2)	C9—H9A	0.9700
C1—C6	1.415 (2)	C9—H9B	0.9700
C2—C3	1.379 (2)	C10—C11	1.532 (2)
C2—H2A	0.9300	C10—C11^i^	1.532 (2)
C3—C4	1.394 (3)	C10—C9^i^	1.539 (2)
C3—H3A	0.9300	C11—H11A	0.9600
C4—C5	1.364 (3)	C11—H11B	0.9600
C4—C7	1.513 (2)	C11—H11C	0.9600
C5—C6	1.421 (2)	O1W—H1	0.9818
			
O1—Ni1—O1^i^	91.34 (6)	C4—C7—H7A	109.5
O1—Ni1—N1^i^	154.58 (6)	C4—C7—H7B	109.5
O1^i^—Ni1—N1^i^	94.14 (5)	H7A—C7—H7B	109.5
O1—Ni1—N1	94.14 (5)	C4—C7—H7C	109.5
O1^i^—Ni1—N1	154.58 (6)	H7A—C7—H7C	109.5
N1^i^—Ni1—N1	91.48 (7)	H7B—C7—H7C	109.5
C1—O1—Ni1	126.63 (9)	N1—C8—C6	125.58 (15)
C8—N1—C9	119.54 (13)	N1—C8—H8A	117.2
C8—N1—Ni1	125.16 (11)	C6—C8—H8A	117.2
C9—N1—Ni1	114.64 (10)	N1—C9—C10	113.57 (13)
O1—C1—C2	118.94 (14)	N1—C9—H9A	108.9
O1—C1—C6	123.87 (14)	C10—C9—H9A	108.9
C2—C1—C6	117.16 (15)	N1—C9—H9B	108.9
C3—C2—C1	121.69 (16)	C10—C9—H9B	108.9
C3—C2—H2A	119.2	H9A—C9—H9B	107.7
C1—C2—H2A	119.2	C11—C10—C11^i^	111.2 (3)
C2—C3—C4	121.84 (17)	C11—C10—C9^i^	106.43 (11)
C2—C3—H3A	119.1	C11^i^—C10—C9^i^	110.61 (11)
C4—C3—H3A	119.1	C11—C10—C9	110.61 (11)
C5—C4—C3	117.16 (16)	C11^i^—C10—C9	106.43 (11)
C5—C4—C7	121.55 (19)	C9^i^—C10—C9	111.63 (18)
C3—C4—C7	121.28 (19)	C10—C11—H11A	109.5
C4—C5—C6	122.98 (17)	C10—C11—H11B	109.5
C4—C5—H5A	118.5	H11A—C11—H11B	109.5
C6—C5—H5A	118.5	C10—C11—H11C	109.5
C1—C6—C5	119.02 (15)	H11A—C11—H11C	109.5
C1—C6—C8	123.49 (15)	H11B—C11—H11C	109.5
C5—C6—C8	117.48 (14)		
			
O1^i^—Ni1—O1—C1	163.48 (15)	C7—C4—C5—C6	178.41 (17)
N1^i^—Ni1—O1—C1	−93.95 (16)	O1—C1—C6—C5	−174.24 (15)
N1—Ni1—O1—C1	8.32 (13)	C2—C1—C6—C5	4.2 (2)
O1—Ni1—N1—C8	1.34 (13)	O1—C1—C6—C8	5.0 (2)
O1^i^—Ni1—N1—C8	−100.61 (16)	C2—C1—C6—C8	−176.58 (15)
N1^i^—Ni1—N1—C8	156.53 (16)	C4—C5—C6—C1	−2.0 (3)
O1—Ni1—N1—C9	171.99 (11)	C4—C5—C6—C8	178.70 (16)
O1^i^—Ni1—N1—C9	70.04 (15)	C9—N1—C8—C6	−177.90 (15)
N1^i^—Ni1—N1—C9	−32.82 (8)	Ni1—N1—C8—C6	−7.7 (2)
Ni1—O1—C1—C2	169.47 (11)	C1—C6—C8—N1	5.6 (3)
Ni1—O1—C1—C6	−12.1 (2)	C5—C6—C8—N1	−175.14 (16)
O1—C1—C2—C3	175.28 (15)	C8—N1—C9—C10	−117.17 (16)
C6—C1—C2—C3	−3.2 (2)	Ni1—N1—C9—C10	71.61 (15)
C1—C2—C3—C4	−0.1 (3)	N1—C9—C10—C11	82.3 (2)
C2—C3—C4—C5	2.4 (3)	N1—C9—C10—C11^i^	−156.78 (16)
C2—C3—C4—C7	−177.33 (18)	N1—C9—C10—C9^i^	−36.00 (9)
C3—C4—C5—C6	−1.3 (3)		

Symmetry codes: (i) −*x*, *y*, −*z*+1/2.

### Hydrogen-bond geometry (Å, °)

**Table:**

*D*—H···*A*	*D*—H	H···*A*	*D*···*A*	*D*—H···*A*
O1W—H1···O1^ii^	0.98	1.92	2.781 (2)	145
C3—H3A···O1W^iii^	0.93	2.55	3.477 (2)	173

Symmetry codes: (ii) −*x*, *y*, −*z*+1/2; (iii) −*x*+1/2, −*y*+3/2, −*z*+1.
